# A Nonsynonymous Substitution of *Lhx3* Leads to Changes in Body Size in Dogs and Mice

**DOI:** 10.3390/genes15060739

**Published:** 2024-06-04

**Authors:** Wanyi Dang, Dali Gao, Guangqi Lyu, David M. Irwin, Songyang Shang, Junnan Chen, Junpeng Zhang, Shuyi Zhang, Zhe Wang

**Affiliations:** 1 College of Animal Science and Veterinary Medicine, Shenyang Agricultural University, Shenyang 110866, China; 2 Department of Laboratory Medicine and Pathobiology, University of Toronto, Toronto, ON M5S 1A8, Canada

**Keywords:** *Lhx3*, SNP, body size, dog, transgenic mouse

## Abstract

*Lhx3* is a LIM-homeodomain transcription factor that affects body size in mammals by regulating the secretion of pituitary hormones. Akita, Shiba Inu, and Mame Shiba Inu dogs are Japanese native dog breeds that have different body sizes. To determine whether *Lhx3* plays a role in the differing body sizes of these three dog breeds, we sequenced the *Lhx3* gene in the three breeds, which led to the identification of an SNP in codon 280 (S280N) associated with body size. The allele frequency at this SNP differed significantly between the large Akita and the two kinds of smaller Shiba dogs. To validate the function of this SNP on body size, we introduced this change into the *Lhx3* gene of mice. Homozygous mutant mice (S279N^+/+^) were found to have significantly increased body lengths and weights compared to heterozygous mutant (S279N^+/−^) and wild-type (S279N^−/−^) mice several weeks after weaning. These results demonstrate that a nonsynonymous substitution in *Lhx3* plays an important role in regulating body size in mammals.

## 1. Introduction

Abnormal anterior pituitary function leads to deficiencies in the amounts of hormones secreted by the pituitary gland. Combined pituitary hormone deficiency (CPHD) is characterized by a lack of growth hormone (GH) that is either isolated or combined with decreased levels of other pituitary hormones [1]. The most notable feature of CPHD is the deficiency in GH secretion, which leads to impaired fetal growth, with adults usually displaying a gnomish phenotype [2]. *Lhx3* is a key regulatory factor for the development of the pituitary gland [3]. The *Lhx3* gene is composed of seven coding exons and six introns and is located on chromosome 9q34 in humans [4]. The coding regions of *Lhx3* for the function protein domain are contained in each exon. Exons 1a and 1b code for alternative amino termini for two *Lhx3* isoforms that possess differing abilities to activate pituitary hormone genes. Exons 2 and 3 encode the LIM1 and LIM2 domains, respectively, while exons 4 and 5 encode a homeodomain. Exon 5 also encodes a transactivation domain that specifically directs pituitary gene expression [5]. Deletion and substitution mutations in exons 2, 3, and 5 have been identified that lead to dwarfism due to a deficiency in pituitary hormones [6].

In mice, *Lhx3* is essential for growth and development, with expression of *Lhx3* starting as early as embryonic day 8.5, and by embryonic day 9.5 expression is found in Rathke’s pouch and the closed neural tube [7]. Previous studies have shown that *Lhx3* is necessary for survival, as mice with a homozygous inactivation of *Lhx3* (*Lhx3*^−/−^) die soon after birth [8]. These mice were found to lack the anterior and intermediate lobes of their pituitary glands [9].

**Figure 1:**
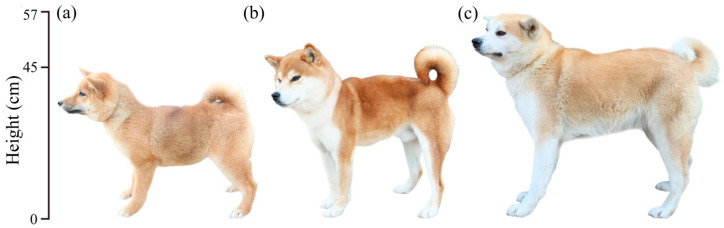
Body size comparison of the three dog breeds in this study. Picture of three types of dogs: (**a**) Mame Shiba Inu, (**b**) Shiba Inu, (**c**) Akita.

Akita, Shiba Inu, and Mame Shiba Inu dogs are all native Japanese dog breeds that have differing body sizes (Figure 1) [10]. The Shiba Inu dog is a small dog breed that was originally used in the Jomon Era as a hunting dog to hunt small animals. In contrast, the Akita dogs are the only large dog breed native to Japan, and purebred animals have been bred in Japan since the Showa era (http://www.nihonken-hozonkai.or.jp/ (accessed on 20 May 2024)). In dogs, the *Lhx3* gene maps to chromosome 9, and spontaneous mutations in this gene have been identified to cause short stature [11]. Dwarfism in dogs is due to spontaneous mutations in *Lhx3* that are often caused by the shortening of a DNA repeat sequence in intron 5 and is often seen in breeds such as the German Shepherd, Dog Salos, and the Czechoslovakian Wolfdog [12]. These mutations typically exhibit autosomal recessive inheritance [11,13,14]. However, to date, the genetic basis for the differences in body size among the three Japanese dog breeds is not known.

In this study, we identified a nonsynonymous substitution (S280N) in the *Lhx3* gene of Akita dogs that may have a positive effect on body size. To confirm this body size effect, we created a transgenic mouse model of this mutation, which showed that this nonsynonymous substitution resulted in transgenic mice with larger body sizes and paralleled the change seen in the Akita dogs.

## 2. Materials and Methods

### 2.1. Animals

All the animal experiments were approved by the Animal Ethics Committee of Shenyang Agricultural University (ID no. 2022030706). There was an equal distribution of male and female numbers among all the experimental animals. A total of 114 dogs (*Canis lupus familiaris*), which included 28 Akitas, 54 Shiba Inus, 32 Mame Shiba Inus, 2 Border Collies, 1 Golden Retriever, and 1 Giant Poodle, were used for this study, and blood samples were collected by venipuncture of their forelimbs. The owners of the dogs provided informed consent for the use of their dogs’ data in this scientific study. All the dogs used in this study were at maturity with ages of 3–6 years and were in good health. Certificates from the Kennel Club of Japan (https://www.kcj.gr.jp/ (accessed on 20 May 2024)), Federation Cynologique Internationale (https://www.fci.be/en/ (accessed on 20 May 2024)), or Japan Kennel Association (https://www.nihonken-hozonkai.or.jp/ (accessed on 20 May 2024)) were obtained for each dog. The body height ranges of the Akita, Shiba Inu, and Mame Shiba Inu dog breeds are listed in Table 1. The wolf blood sample used in this study was collected from a Mongolian wolf (*Canis lupus chanco*) bred in Shenyang Forest Zoological Garden. In this experiment, we used 30 homozygous mice, including 14 males and 16 females, 30 heterozygous mice, including 15 males and 15 females, and 30 wild mice, including 14 males and 16 females. The mice were housed in a specific pathogen-free environment under controlled conditions of temperature and light (12 h on/12 h off) and were provided free access to water and commercial mouse chow. The homozygous mice used in the experiment were the 4th generation, and the heterozygous mice were the 4–6th generation.

### 2.2. DNA Extraction, PCR Amplification, Sequence Analysis, and Protein Structure Prediction

Genomic DNAs were extracted from the dogs, the wolf blood sample, and a small piece of amputated mouse finger using the TIANamp Genomic DNA Kit (Tiangen Biotech, Beijing, China) according to the manufacturer’s instructions, except that the time for the lysis step was reduced from 30 min to an hour. The concentration of DNA was determined from OD260 values using a NanoDrop 2000 (Thermo Fisher Scientific, Waltham, MA, USA).

To determine *Lhx3* genotypes, primers were designed using Primer premier v5 to amplify the *Lhx3* coding region [15]. To identify the *Lhx3* sequence in the wolf, the primer sequences *Lhx3w*-forward 5′-GGTCAACCTCATCCAGCCAT-3′ and *Lhx3w*-reverse 5′-CTGCCAACGGCCTCTAC-3′ were used. For the identification of *Lhx3* in the dogs, the primer sequences *Lhx3d*-forward 5′-GGAGACTCTGACTGCATTGTGAC-3′ and *Lhx3*d-reverse 5′-CCAAGCTGGCATCTGGATATAC-3′ were used. For the mice, the primer sequences *Lhx3m*-forward 5′-AGGCTCAAGTTGGTGTCTG-3′ and *Lhx3m*-reverse 5′-CACTCCACTACCCACAGCC-3′ were used. PCR reactions were performed using a C1000 thermal cycler (Bio-Rad Laboratories Inc., Hercules, CA, USA) in a total volume of 30 μL containing 10–50 ng genomic DNA, 0.5 µL of each primer, 15 μL 2 × EasyTaq PCR Supermix (TransGen Biotech Co., Beijing, China), and 13 μL ddH_2_O. The conditions for *Lhx3* amplification were an initial denaturation at 95 °C for 5 min, followed by 30 cycles of denaturing at 95 °C for 30 s, annealing at 58 °C for 30 s, and extension at 72 °C for 30 s, with a final extension at 72 °C for 10 min. The products from the PCR amplifications were purified and sequenced by the Sanger method. The *Lhx3* coding region of the species mentioned in the article is entirely derived from http://www.ensembl.org/ (accessed on 20 May 2024) and https://www.ncbi.nlm.nih.gov/ (accessed on 20 May 2024).

The *Lhx3* protein sequences were aligned by MUSCLE using MEGA 11.0 [16]. The Expasy (Expasy–ProtParam tool) was used to examine the impact of amino acid substitutions on the *Lhx3* protein secondary structure [17]. The phylogenies were performed using the maximum likelihood (ML) method based on the Tamura–Nei model [18]. Statistical support was assessed with bootstrap analysis (1000 replicates) [19].

### 2.3. Generation of Transgenic Mouse with Point Mutation

A mouse model with the S279N substitution in mouse *Lhx3* was generated using CRISPR/Cas-mediated genome engineering [20]. The mouse *Lhx3* gene is located on chromosome 2, and the targeted mutation (S279N) is located within exon 6. The gRNA targeting vector and donor oligomers were based on the target sequences in the mouse. The donor oligo was composed of 120 bp of homologous sequence that flanked the S279N (AGC to AAC) mutation site. The mutation was introduced into exon 6 using a donor oligonucleotide and Cas9-directed repair. The gRNA generated was by in vitro transcription, and the gRNA together with the donor oligo were co-injected into fertilized eggs for knock-in (KI) mouse production [21]. The target region of the mouse *Lhx3* locus was amplified by PCR with specific primers (*Lhx3m*-forward 5′-AGGCTCAAGTTGGTGTCTG-3′ and *Lhx3m*-reverse 5′-CACTCCACTACCCACAGCC-3′), and the size of the product was 788 bp. The PCR products were sequenced to confirm the targeting locus (Figure S1).

### 2.4. Significance Analysis

The genotyping data of the Akita, Shiba Inu, and Mame Shiba Inu dogs were analyzed; the independence test in the chi square test was conducted using R (version 3.6.1) statistical language; and the theoretical expected frequency of AA, AG, and GG for each genotype accounted for one-third of each variety. Significance is reported at *p* ≤ 0.05. To investigate the effect of the S279N *Lhx3* mutation on body size in mice, we collected body length and weight data from individual mice between the ages of 4 weeks (w) and 12 w after birth for mice with the three different *Lhx3* genotypes. Body length and weight data were imported into SPSS (version 21) for a one-way analysis of variance (ANOVA). The data were reported as means ± SEM in the text and presented as a line chart with asterisks representing significance. Significance is reported at *p* ≤ 0.05.

## 3. Results

### 3.1. Identification of a S280N Replacement Mutation in Large-Sized Japanese Dogs

To better understand the genetic basis for the size variation in Japanese dogs, we sequenced the whole coding region of the *Lhx3* gene from 1 Mongolian wolf, 28 Akita (large body size), 54 Shiba Inu, and 32 Mame Shiba Inu dogs (small body size). The wolf was included as the outgroup to identify the direction of mutational changes. From these sequence data, we identified a nonsynonymous substitution at the 839th nucleotide of the *Lhx3* coding sequence that results in a serine (AGC) to asparagine (AAC) replacement (S280N). The S280N replacement was at high frequency in the large-sized Akita dog sequences and nearly absent in the sequences from the wolf and the small-sized Shiba Inu and Mame Shiba Inu dogs.

The frequency of the base at this SNP differed greatly; the χ^2^ text results show that the phenotypes of body size were significantly related with the SNP locus (S280N) in *Lhx3* (*p* < 0.01) between the Akita dog and the two kinds of Shiba Inu dogs. For the Akita dog, 25 of the 28 (89.3%) Akita dogs had the A/A (Asn) genotype, while 47 of the 54 Shiba Inu dogs (87.0%) and 26 of the 32 (81.3%) Mame Shiba Inu dogs had the G/G (Ser) genotype at this SNP (Table 1). The remaining dogs from these three examined breeds were heterozygous, having both an A and a G allele at this SNP. As the Mongolian wolf had the G/G genotype at the SNP, this suggests that it was the ancestral genotype and that the A allele (and asparagine codon) was gained by the Akita dogs.

**Figure 2:**
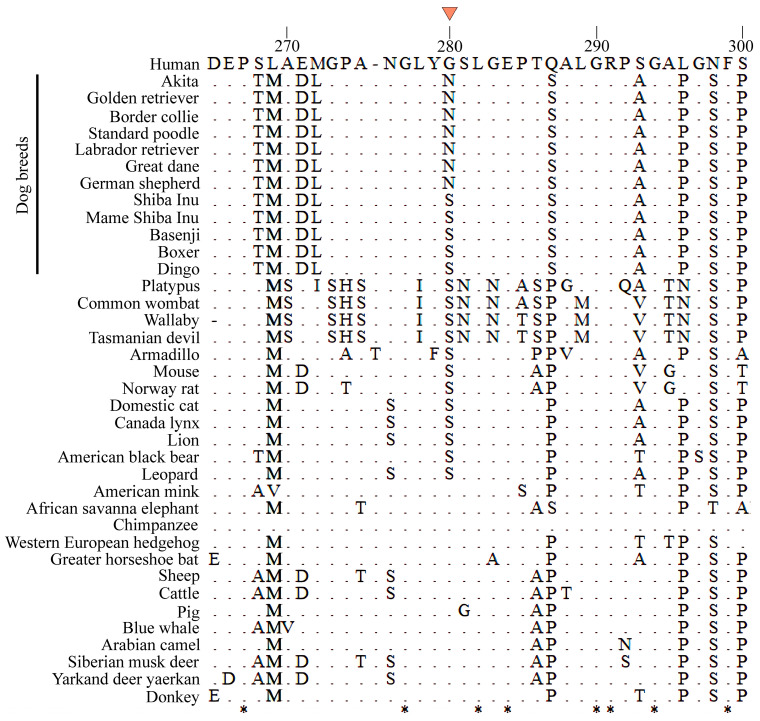
Alignment of part of LHX3 from diverse mammals that contains the amino acid substitution (at amino acid site 280) associated with body size. The triangle above the alignment indicates the polymorphic site. * the amino acid at this site is identical in the species.

**Figure 3:**
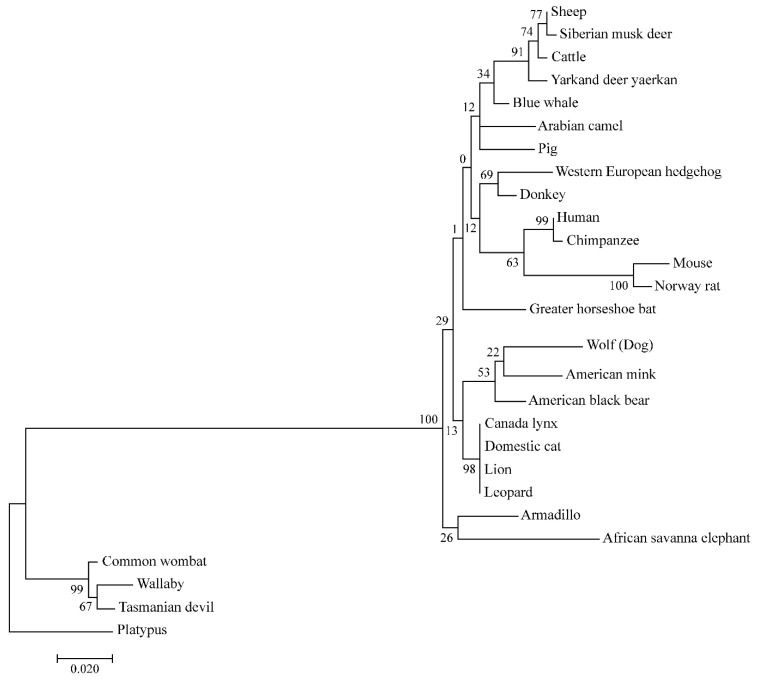
ML phylogenetic analysis of partial *Lhx3* protein sequences from diverse mammals. The tree was rooted using the sequence from a monotreme, the platypus. Bootstrapping (1000 replications) was used to assess support for the clustering of taxonomic groups, and support levels are shown next to the branches. Branch lengths are scaled to the number of substitutions per site.

Examination of the *Lhx3* coding sequences in other dog breeds found an asparagine (AAC) codon at residue 280 in the sequences from the large-sized breeds (Labrador retriever, Great Dane, German Shepherd, Golden retriever, Border collie and Standard poodle) and a serine (AGC) codon from the smaller-sized breeds (Basenji, Boxer, and Australian dingo). Thus, Asn280 is associated with larger body size and Ser280 with smaller body size in dogs (Figure 2). When Expasy was used to predict the consequence of this substitution for the LHX3 protein secondary structure, we found the S279N replacement caused an increase in the stability coefficient for the secondary structure to 67.39 from 57.10. When the LHX3 sequences of other mammals were examined, the residue homologous to position 280 in the dog sequence was found to be glycine in the larger-sized mammals, such as humans, elephants, blue whales, and cattle, and, interestingly, serine, like the small dogs, in the small-sized cat and mouse (Figure 2). The evolutionary relationships of these species (Figure 3), based on an ML phylogenetic analysis of the partial *Lhx3* protein sequences, is consistent with their expected relationships.

### 3.2. Influence of the S280N Substitution on Body Size in Mice

**Figure 4:**
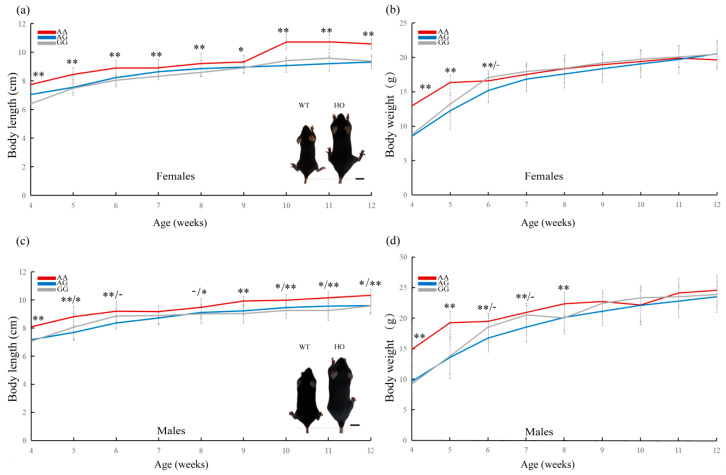
Analysis of body length and weight in *Lhx3* mutant mice. Homozygous (S279N^+/+^) female (**a**,**b**) and male (**c**,**d**) mice have longer body lengths and greater body weights than heterozygous (S279N^+/−^) and wild-type (S279N^−/−^) mice at certain weeks. Bars represent mean ± SEM. The asterisk represents the significance of comparison between homozygous and heterozygous mice (before the slash) or wild-type mice (after the slash). If they are the same, there is no slash. One asterisk indicates *p* < 0.05, two asterisks indicate *p* < 0.01, and a dash indicates no significance. Inset shows wild-type (left) and homozygous (right) mice at 4 weeks old.

To test the effect of the S280N substitution on body size in mammals, we generated knock-in mutant mice using CRISPR-Cas9 on the equivalent position (S279N). The *Lhx3* gene of mice has serine at codon 279; thus, replacing the serine codon with asparagine will mimic the S280N replacement seen in larger dog breeds, including the Japanese Akita dog. We found that homozygous mutant mice (S279N^+/+^) basically had significantly longer body lengths than heterozygous (S279N^+/−^) and wild-type (S279N^−/−^) mice from the ages of 4 to 12 weeks in both genders (Figure 4a,c). No significant difference in body length was seen between the heterozygous and wild-type mice at these times. We selected the fourth-week homozygous (S279N^+/+^) and wild mice (S279N^−/−^) with the largest difference in body length data to create a comparison chart (Figure 4a,c). For the females, the homozygous mutant mice (S279N^+/+^) basically had significantly greater body weights than the heterozygous (S279N^+/−^) and wild-type (S279N^−/−^) mice from the ages of 4 to 6 weeks (Figure 4b). For the males, the homozygous mutant mice (S279N^+/+^) basically had significantly greater body weights than the heterozygous (S279N^+/−^) and wild-type (S279N^−/−^) mice from the ages of 4 to 8 weeks (Figure 4d). These results suggest that the S279N replacement affects the regulation of body size, especially body length. The lack of an effect of the substitution on body weight at older ages may be due to compensatory effects by other genes contributing to body weight.

## 4. Discussion

LHX3 is a marker of early anterior pituitary development [22,23,24]. The deletion of this gene leads to an incomplete development of the pituitary gland, resulting in CPHD in both humans and dogs [12,22,23,24,25]. Individuals with CPHD caused by an *Lhx3* mutation are deficient in GH and other essential hormones, resulting in short stature [26,27]. Previous studies have described mutations in *Lhx3*, such as the insertion of a premature stop codon, which leads to a deletion of the carboxyl terminus of the protein, a region that contains activation/repression functions and modification/targeting signals, which leads to short stature [28]. Patients with LHX3 (E173Ter) mutation also have CPHD diseases featuring losses of GH and other essential hormones; in addition, the mutation also affects both the amino and carboxyl termini of the protein or disables the protein by affecting the DNA-binding domain [29]. Unlike the previous studies, here we found an LHX3 amino acid replacement (S280N) that has a positive effect on somatic growth. The S280N nonsynonymous substitution in LHX3 might cause animals to become larger due to the stabilization of the protein secondary structure. As shown above, an analysis by Expasy indicated that LHX3 with N280 had a higher stability and therefore a potentially longer half-life than LHX3 with Ser280. This amino acid substitution in *Lhx3* is associated with similar manifestations of body size in both dogs and mice, as shown by our results with transgenic mice. Since the A/A genotype was not found in the smaller Japanese dog breeds (Shiba Inu and Mame Shiba Inu dogs that have differing heights), this indicates that the S280N substitution in LHX3 is not the only factor affecting body size in the Japanese dog breeds but is an important factor for the increased size of the Akita dog. We detected the A/G genotype in several individuals of Akita, Shiba Inu, and Mame Shiba Inu dogs, which indicates that the *Lhx3* locus does not completely determine their body sizes and that other loci or genes are involved. A previous study found that seven markers from *GHR*, *HMGA2*, *SMAD2*, *STC2*, *IGF1*, and *IGF1R* explain 46% to 52.5% of the variation in body size in small dog breeds [30]. Mutations in antisense long non-coding RNA (*IGF1-AS*), which can interact with *IGF1* transcripts to create a duplex, are associated with body size in large dogs [31]. In addition to the *Lhx3* locus investigated in this study, these other genes and loci likely also participate in the regulation of the body size difference between the Akita, Shiba Inu, and Mame Shiba Inu dog breeds.

The Mame Shiba Inu dogs were recently derived from the Shiba Inu dog breed and were recognized as a breed by the Kennel Club of Japan as well as a unique breed in both Japan and China [32]. It appears that the Shiba Inu dogs were bred, independently of other world-wide dog breeds, from the wolf in about 1000 BC [33]. Through a comparison of *Lhx3* sequences from different breeds of dogs, we found that the same S280N replacement found in the Akita dog also appears in the large-sized dogs, but not in the smaller-sized Basenji, Boxer, and Australian dingo. Moreover, we did not find the same amino acid substitution (S280N) at this position in LHX3 of other mammals. The G/G (Ser280) genotype in dogs was likely derived from their wild ancestors as the wolf also has this genotype. Shiba Inu dogs would then have acquired the mutated A/G genotype. As the Shiba Inu dogs have a smaller body size than the wolf, this could be due to changes in other genes. The larger Akita dogs were bred from the Shiba Inu dog lineage in the 17th century through acquiring the A/A genotype for *Lhx3* at codon 280, in similar fashion as the origin of the Labrador retriever from its small-body forebear, the St. John’s water dog in Newfoundland [34]. As the A/A genotype at this SNP location is associated with larger body size, as shown with our transgenic mice, selection for larger body size may have driven the increase in this genotype in parallel in multiple mammalian lineages, not only in the Japanese Akita dog.

We identified a new SNP in *Lhx3* that might affect body size in mammals by changing the stability of the LHX3 protein. However, the specific consequence of the substitution for the regulatory mechanisms that control body size remain unclear, and further research is required. In future work, we will examine the effect of the *Lhx3* gene mutation in mice on hormonal changes (such as GH, IGF-I, and thyroxine) and analyze transcriptome data from various weight-related tissues (e.g., adipose tissue and muscle) from mutant and wild-type mice, to better understand the mechanisms responsible for the changes in body size.

## Figures and Tables

**Table 1:** Genotype frequency of SNP (S280N) in Akita, Shiba Inu, and Mame Shiba Inu dogs.

Breed	Body Height	Total	G/G	A/G	A/A	χ^2^ Value
Akita	58–70 cm	28	0	3	25	
Shiba Inu	35–41 cm	54	47	7	0	χ^2^ = 101.43 *p* < 0.01
Mame Shiba Inu	25–32 cm	32	26	6	0	

Total represents the total number of genotypes for the three breeds of dogs. G/G, wild type; A/G, heterozygous type; A/A, homozygous type. Ranges of body height for Akita and Shiba Inu were from the official websites issuing their certificates (https://www.nihonken-hozonkai.or.jp/akitaken/ (accessed on 20 May 2024) and https://www.nihonken-hozonkai.or.jp/shibainu/ (accessed on 20 May 2024)). The height range for the Mame Shiba Inu was from their certificates.

## Data Availability

The original contributions presented in the study are included in the article and Supplementary Materials, further inquiries can be directed to the corresponding author.

## Supplementary Materials

The following supporting information can be downloaded at: https://www.mdpi.com/article/10.3390/genes15060739/s1, Figure S1: Sanger sequencing of the *Lhx3* mutation site in mice.
