# Supplementary material for: A Nonsynonymous Substitution of Lhx3 Leads to Changes in Body Size in Dogs and Mice
Source: Genes (Basel). 2024 Jun 4;15(6):739. doi: 10.3390/genes15060739 (PMC11202965; doi:10.3390/genes15060739)
Supplement: Supplementary file 1 [file genes-15-00739-s001.zip › genes-3025204-supplementary.pdf]

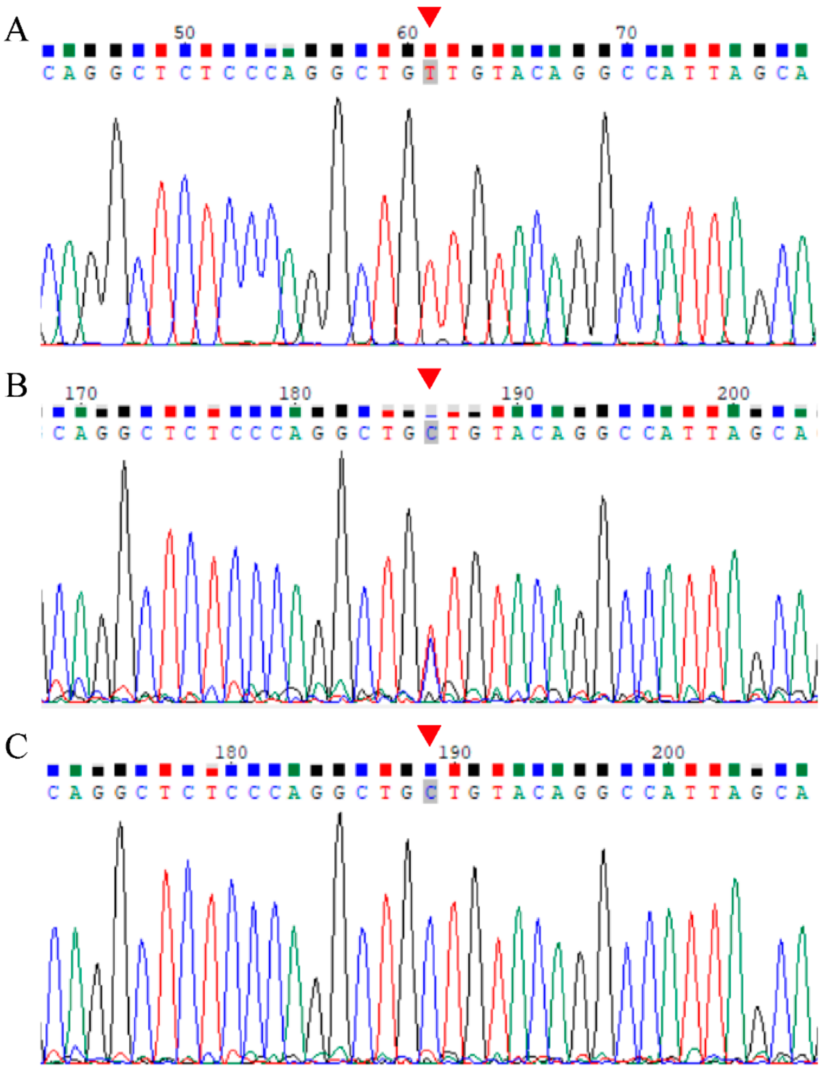

Figure S1. Sanger sequencing of the *Lhx3* mutation site in mice. (A–C) Sanger sequencing chromatograms from (A) homozygous (S279N$^{+/+}$), (B) heterozygous (S279N$^{+/-}$) and (C) wild-type (S279N$^{-/-}$) mice. The strand complementary to the coding sequence is shown. Red arrows indicate the mutation site.
